# Perceived Self-Control Effort, Subjective Vitality, and General Affect in an Associative Structure

**DOI:** 10.3389/fpsyg.2021.575357

**Published:** 2021-04-14

**Authors:** Alex Bertrams

**Affiliations:** Educational Psychology Lab, Institute of Educational Science, University of Bern, Bern, Switzerland

**Keywords:** affect, effort, ego depletion, energy, fatigue, self-control, self-regulation, subjective vitality

## Abstract

A crucial assumption of the recently developed *schema model of self-control* is that people’s perceived self-control efforts are related to the experience of lowered subjective vitality. In the present study, this assumption was tested. It was also examined whether perceived self-control effort is related to a diffuse affective experience (i.e., subjective vitality, general positive affect, and general negative affect as a combined factor) or is discretely related to subjective vitality, general positive affect, and general negative affect. Based on the previous literature, it was expected that the latter would better fit the data. In a survey study, university students (*N* = 501) completed standardized measures of their perceived self-control effort, subjective vitality, general positive affect, and general negative affect with regard to a specific frame of reference (i.e., during the current day and the last 2 days). Bivariate correlations and confirmatory factor analyses revealed the expected relationships, meaning that perceived self-control effort was negatively related to subjective vitality and that the statistical model with three distinct affective variables fit the data better than the model with subjective vitality, positive affect, and negative affect incorporated into one common factor. It was concluded that the findings are in line with the schema model of self-control.

## Introduction

The human ability to exert self-control is crucial for positive functioning (Tangney et al., 2004; Baumeister and Vohs, 2016). In this regard, *self-control* is defined as the process of overriding or altering one’s dominant response tendencies (Muraven and Baumeister, 2000; Bertrams et al., 2016). For instance, students exert self-control when they suppress the impulse to have fun with their friends at their favorite pub and instead study for upcoming exams. Baumeister et al. (1994) categorized the forms of self-control into controlling one’s impulses, thoughts, emotions, and task-related behaviors. Despite the importance of self-control for adjustment, people frequently fail to control themselves (Baumeister et al., 1994). One reason may be that the demanded self-control effort is often accompanied by the unpleasant subjective experience of energy loss (i.e., decreased subjective vitality), which reduces the motivation to exert further self-control (Bertrams, 2020). In some studies, the demanded short-term exertion of self-control in the laboratory has been found to diminish subjective vitality (Muraven et al., 2008; Legault et al., 2009), and the intensity of self-control efforts over the course of the day was associated with reduced subjective vitality later in the day (van Hooff and Geurts, 2015; Gombert et al., 2020). Moreover, many studies have shown that self-control efforts are followed by self-perceived fatigue (Hagger et al., 2010; van Hooff and Geurts, 2015), whereby subjective fatigue can be considered the opposite of subjective vitality (Ware and Sherbourne, 1992; Deng et al., 2015).

*Subjective vitality* is defined as the positively toned self-perception of having available energy and feeling alive (Ryan and Frederick, 1997) and is not necessarily related to physiological energy (Martela et al., 2016). In accordance with its role in individuals’ healthy functioning and wellbeing (Ryan and Deci, 2001), higher subjective vitality is substantially related to higher emotional wellbeing in terms of general affect (e.g., Martela and Ryan, 2016; Bertrams et al., 2020); however, subjective vitality has been theoretically and empirically distinguished from measures of general positive and negative affect (Ryan and Frederick, 1997; Nix et al., 1999; Bertrams et al., 2020). Thus, subjective vitality may not be redundant to general affect (i.e., feeling globally positive or globally negative) and may be a variable in its own right.

**FIGURE 1:**
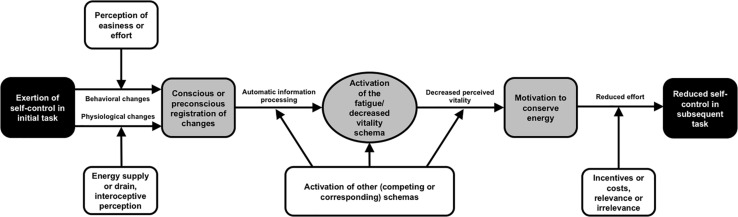
The schema model of self-control (Figure taken from Bertrams, 2020). Black boxes: the observable behavior in self-control studies. Gray boxes and horizontal arrows: the mediating processes within the individual. White boxes: moderating variables.

At its core, the recently developed *schema model of self-control* (Bertrams, 2020; see Figure 1) posits that individuals’ exertion of effortful self-control releases the schematic activation of decreased subjective vitality. One way by which the schema of decreased vitality is assumed to be activated within individuals is the self-perception of engaging in effortful behavior. This means that individuals’ perceived self-control effort and their subjective vitality should be associated in a cognitive structure. Thus, the model predicts a negative relationship between perceived self-control effort and experienced subjective vitality, which is in line with previous findings (van Hooff and Geurts, 2015; Gombert et al., 2020). According to the schema model, the experience of decreased vitality can remain preconscious when the self-control demands have a low intensity; however, following intense self-control demands, people should become aware of their self-control efforts and, consequently, of their lowered subjective vitality and should thus be able to report it in self-report measures.

Because the experience of decreased subjective vitality is central in the schema model of self-control, it is important for this model that subjective vitality is a distinct variable within the association between perceived self-control effort and subjective vitality. Therefore, the model is challenged by the fact that subjective vitality is generally substantially correlated with measures of general positive and negative affect (e.g., Bertrams et al., 2020). If perceived self-control effort releases a diffuse experiential blend of lowered subjective vitality and general affect, the decisive informational value of subjective vitality within the schema model of self-control would be questioned. In other words, the core of the schema model is that perceived self-control effort is associated with the perceived loss of energy and aliveness (i.e., the decrease in subjective vitality) rather than with just feeling less well or worse. This distinction is relevant, as it is argued in the schema model of self-control that from perceived energy loss logically follows the motivation to save energy and therefore to avoid further behaviors that are perceived as energy-costly, such as self-control.

In laboratory studies, brief self-control demands perceived as effortful have been shown to have an effect on subjective vitality, whereas such brief demands did not influence general affect (Muraven et al., 2008; Legault et al., 2009). This pattern indicates the differentiability of subjective vitality and general affect; however, the pattern is not as clear for cumulated self-control efforts (e.g., over several days). Studies from the realm of occupational psychology showed that the more intensely employees rated their demanded self-control efforts at work, the lower they estimated their subjective vitality, but also the worse they felt in terms of affective measures beyond their subjective energy levels (Schmidt and Neubach, 2010; Schmidt and Diestel, 2014). Thus, it could be that for longer-lasting self-control demands, subjective vitality is confounded with general affect in the cognitive association between perceived self-control effort and subjective vitality. However, from the perspective of the schema model of self-control, perceived self-control efforts reduce the experience of subjective vitality, which leads to lowered engagement in self-control in terms of thought control. Low thought control then prevents individuals from generating a pleasant stream of thoughts, causing reduced pleasant affect (He et al., 2019). Thus, although related, perceived self-control effort, subjective vitality, and general affect should be conceptually and empirically distinguishable from each other.

In the present study, the aim was to clarify the associative structure of perceived self-control effort, subjective vitality, and general affect. For this purpose, a survey was administered to university students during the middle of the ongoing semester, as the individually different demands of academic learning at this time should provide sufficient variance in the momentary perceived self-control effort. The hypotheses were based on the schema model of self-control (Bertrams, 2020), as well as the theory and empirical findings that indicate that subjective vitality is distinct from general affect (Ryan and Frederick, 1997; Bertrams et al., 2020) and that even positive affect and negative affect are distinct from each other (Watson et al., 1988; Rahm et al., 2017). It was predicted that perceived self-control effort is negatively related to subjective vitality. Moreover, it was assumed that a confirmatory factor analysis would reveal a superior model when subjective vitality, positive affect, and negative affect were modeled as distinct factors, as opposed to being merged within a combined factor of affective experience.

## Materials and Methods

### Participants

The participants (*N* = 501 university students from various subjects; 74% female; *M*_age_ = 22.07, SD_age_ = 2.86) were approached in the buildings of two universities in the German-speaking part of Switzerland. They were asked to complete a brief questionnaire. One participant did not complete the individual-difference-level scale of the Subjective Vitality Scales-German and is therefore not included in the respective analysis. All participants indicated that they understood the written German language well.

### Measures

The questionnaire consisted of questions on socio-demographic variables (e.g., age) and the standardized and validated scales described in the following.

#### Perceived Self-Control Effort

This variable was measured with the subscales *overcoming inner resistances* (five items; e.g., “Tackling certain tasks sometimes costs/cost me a lot of effort”) and *resisting distractions* (four items; e.g., “My duties require/required me to ignore distractions as much as possible”) from Schmidt and Neubach’s (2010) German self-control demands scale. According to van Hooff and Geurts (2015), this instrument captures perceived self-control effort, as it not only measures externally generated demands posed upon the respondent but also an internal process within the respondent. The verbs within the items were supplemented with the respective past tense, as the participants were instructed to refer their answers to the current day and the last two days. This temporally expanded frame of reference should ensure that the measurement would less be biased by a momentary brief period without self-control exertion within a longer-lasting intensive self-control phase. The students were also told that the items referred to their study work, as well as any other possible demands, such as employment, household chores, or childcare. The items were completed on response scales ranging from 1 (*not true at all*) to 5 (*very true*). (Note that the subscale *impulse control* from Schmidt and Neubach’s measure was not applied, as it did not fit the student work and life context).

#### Subjective Vitality

The state-level and the individual-difference-level scale of the Subjective Vitality Scales (SVS; Ryan and Frederick, 1997) were used in the German adaptation (SVS-G; Bertrams et al., 2020). In line with Bertrams et al.’s (2020) findings, each scale consisted of five items, each of which was answered on a response scale from 1 (*not at all true*) to 7 (*very true*). In accordance with Ryan and Frederick (1997), the participants were instructed to indicate how much the statement applied to them that day and the last two days (state-level scale) and the degree to which the statement is true for them in general in their lives (individual-difference-level scale). Sample items include “At this time, I feel alive and vital” (state-level scale) and “I feel alive and vital” (individual-difference-level scale), respectively. For the present study, the state-level scale was of central relevance.

#### General Affect

General positive affect (six items; e.g., “good”) and negative affect (six items; e.g., “bad”) were measured using the German adaptation (Rahm et al., 2017) of the Scale of Positive and Negative Experience (SPANE; Diener et al., 2010). The participants were asked to indicate how often during that day and the last two days they had felt the way described by the respective adjective. The response scales ranged from 1 (*very rarely or never*) to 5 (*very often or always*).

## Results

The means, standard deviations, and McDonald’s omegas of the total scores of the applied measures, as well as their intercorrelations, are presented in Table 1. As can be observed, all variables were significantly correlated with each other (except for age and gender). This includes the expected negative correlation between perceived self-control effort and subjective vitality. A paired-samples *t*-test showed that the participants’ momentary subjective vitality (state-level) was lower than in general in their lives (individual-difference-level); *t*(499) = 20.04, *p* < 0.001, *r*_between measures_ = 0.40, *d*_*z*_ = 0.90.

**TABLE 1:** Descriptive statistics and intercorrelations of the applied measures.

Measure	ω	*M*	SD	Intercorrelations						
				1	2	3	4	5	6	7
1. Self-control effort – overcoming inner resistances	0.86	3.41	0.81	–						
2. Self-control effort – resisting distractions	0.81	3.47	0.82	0.29***	–					
3. Subjective vitality – state level	0.90	3.99	1.21	−0.22***	−0.13**	–				
4. Subjective vitality – individual difference level	0.84	5.07	0.94	−0.12**	−0.09*	0.40***	–			
5. Positive affect	0.89	3.67	0.67	−0.22***	−0.15***	0.64***	0.37***	–		
6. Negative affect	0.80	2.33	0.69	0.27***	0.15***	−0.49***	−0.24***	−0.65***	–	
7. Age	–	22.07	2.86	−0.16***	–0.06	0.12**	0.07	0.01	–0.03	–
8. Gender	–	–	–	–0.05	0.07	0.14**	–0.02	0.02	−0.10*	0.04

*N* = 501 (*N* = 500 for subjective vitality – individual difference level). Overall scores of a psychometric scale were obtained by averaging the responses to the scale items. Coding for gender: 1 = female, 2 = male. *p < 0.05. **p < 0.01. ***p < 0.001.

**FIGURE 2:**
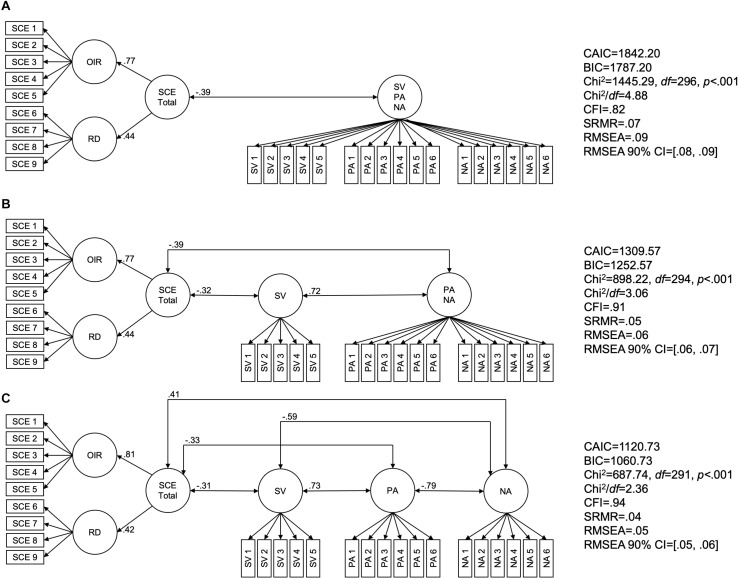
Model tests applying confirmatory factor analyses with AMOS 26. Maximum likelihood estimation was applied. Depicted are the standardized regression weights and correlations, which were all significant at *p* < 0.001. **(A)** Subjective vitality, positive affect, and negative affect are combined within one common factor (i.e., as a completely undifferentiated affective experience). **(B)** Subjective vitality is separated from undifferentiated positive and negative affect. **(C)** Subjective vitality, positive affect, and negative affect are treated as three separate factors (i.e., as three distinguishable affective experiences). OIR, overcoming inner resistances (facet of perceived self-control effort); RD, resisting distractions (facet of perceived self-control effort); SCE Total, perceived self-control effort in total (second-order factor); SV, subjective vitality; PA, positive affect; NA, negative affect; CAIC, consistent Akaike information criterion; BIC, Bayesian information criterion; CFI, comparative fit index; SRMR, standardized root mean square residual; RMSEA, root mean square error of approximation; CI, confidence interval for RMSEA. *N* = 501.

Three factor models were compared with the variables perceived self-control effort (represented by its two related facets, *overcoming inner resistances* and *resisting distractions*), subjective vitality, positive affect, and negative affect (all variables measured with respect to that day and the last two days as the frame of reference). A model with a lower consistent Akaike information criterion (CAIC) and Bayesian information criterion (BIC) was considered superior to another model, whereby a difference in CAIC or BIC of 10 or more is strong evidence for one model over the other (Dziak et al., 2020). The models, as well as all relevant parameters, are depicted in Figure 2.

The first model (see Panel A) suggested that the participants would not have distinguished the subjective experiences of subjective vitality, positive affect, and negative affect from each other. This model is not in line with the literature and revealed a lower CAIC and BIC than the other two models (see Panels B and C). In the second model (see Panel B), subjective vitality was treated as an experiential variable of its own rather than merged with global affect, whereas positive and negative affect were still combined within one common affect factor. The substantial decreases in the CAIC and the BIC clearly indicated the superiority of this second model over the first model. The additional separating of positive affect and negative affect from each other in the third model (see Panel C) yielded a further substantial decrease in the CAIC and the BIC, which meant that this model was preferable over the other two. In the second and third models, perceived self-control effort and subjective vitality were expectedly negatively related (see panels B and C).

## Discussion

In the present study, the relationship between perceived self-control effort and the subjective experience of vitality was examined. For this purpose, a survey was administered to students who were in a situation that, on average, elicited the experience of lowered subjective vitality in comparison to their usual vitality levels (as indicated by the difference between subjective vitality on the state- and individual-difference-levels). Therefore, in the present sample, some individuals had lowered subjective vitality that could be explained by another variable, such as perceived self-control effort.

Conforming with the predictions, a higher perceived self-control effort was associated with lower subjective vitality. Moreover, there was an associative structure in which the relationship between perceived self-control effort and subjective vitality was distinguishable from general positive affect and general negative affect. These findings are in line with the recently developed schema model of self-control (Bertrams, 2020). The implication is that the schema model of self-control was not falsified and should be further empirically tested. While supporting the schema model, the present findings do not contradict other relevant theoretical accounts (e.g., the strength model of self-control; Baumeister and Vohs, 2016); however, the purpose of the present study was not to test different self-control theories against each other but to test the schema model. Future research could be conducted to compare differing self-control theories with regard to their usefulness.

Some limitations of the present work should be mentioned. The study strongly focused on one segregated aspect of the schema model of self-control, namely, the association between perceived self-control effort and subjective vitality. While this relationship is essential in the schema model of self-control, other important relationships assumed in the model have not been examined. Therefore, the present study may not be viewed as a comprehensive test of the schema model of self-control as a whole. Moreover, the causality in the relationships between the variables of interest was not considered. According to the schema model, perceived self-control effort should *cause* a decrease in subjective vitality. It could also be predicted that lowered subjective vitality would *lead to* a decrease in positive affect and an increase in negative affect via diminishing the motivation for subsequent self-control (e.g., controlling one’s stream of thought such that it elicits more positive feelings; He et al., 2019); however, the scope of the present study was the conceptual and empirical distinction between the examined variables to test one sharply outlined key premise of a theoretical model. For this reason, it was sufficient to conduct a cross-sectional correlational study.

Future research could delve deeper into the question of causal relationships by applying experiments or longitudinal designs. It could also include an examination of the preconscious processes hypothesized in the schema model of self-control (see Bertrams, 2020). The present study does not offer insight into such processes. Further research may also take a close look at moderating processes that play an important role at several points within the schema model of self-control (see the white boxes in Figure 1). For instance, people’s implicit theories of whether willpower is a limited or an unlimited resource (Job et al., 2010; Compagnoni et al., 2020) can influence how perceived self-control demands are related to affective experiences (Konze et al., 2018). Another relevant moderating influence may be the extent to which individuals feel autonomous during a self-control demand. With their work based on self-determination theory (Deci and Ryan, 1985; Ryan and Frederick, 1997), Nix et al. (1999) found autonomous motivation during tasks to be vitalizing. Thus, autonomy should counteract or override the schematic activation of decreased subjective vitality induced by perceived self-control effort.

## Data Availability Statement

The raw data supporting the conclusions of this article will be made available by the author, without undue reservation.

## Ethics Statement

The studies involving human participants were reviewed and approved by the Institutional Review Board of the Faculty of Human Sciences at the University of Bern. The patients/participants provided their written informed consent to participate in this study.

## Author Contributions

The author confirms being the sole contributor of this work and has approved it for publication.

## Conflict of Interest

The author declares that the research was conducted in the absence of any commercial or financial relationships that could be construed as a potential conflict of interest.
